# Early fluid management affects short-term mortality in patients with end-stage kidney disease undergoing chronic hemodialysis and requiring continuous renal replacement therapy

**DOI:** 10.1186/s12882-022-02725-7

**Published:** 2022-03-14

**Authors:** Kyun Young Kim, Jung-Hwa Ryu, Duk-Hee Kang, Seung-Jung Kim, Kyu Bok Choi, Shina Lee

**Affiliations:** Department of Internal Medicine, School of Medicine, Ewha Womans University, Seoul, Korea

**Keywords:** Cumulative input, Cumulative output, Short-term mortality, Chronic hemodialysis, Continuous renal replacement therapy

## Abstract

**Background:**

Early fluid management is considered a key element affecting mortality in critically ill patients requiring continuous renal replacement therapy (CRRT). Most studies have primarily focused on patients with intrinsic acute kidney injury requiring CRRT, although end-stage kidney disease (ESKD) patients generally exhibit greater vulnerability. We investigated the association between fluid balance and short-term mortality outcomes in ESKD patients undergoing chronic hemodialysis and requiring CRRT.

**Methods:**

This retrospective study included 110 chronic hemodialysis patients who received CRRT between 2017 and 2019 at Ewha Womans University Mokdong Hospital. The amounts of daily input and output, and cumulative 3-day and 7-day input and output, were assessed from the initiation of CRRT. The participants were classified into two groups based on 7-day and 14-day mortalities. Cox regression analyses were carried out on the basis of the amounts of daily input and output, cumulative input and output, and cumulative fluid balance.

**Results:**

During follow-up, 7-day and 14-day mortalities were observed in 24 (21.8%) and 34 (30.9%) patients. The patients were stratified into two groups (14-day survivors vs. non-survivors), and there were no significant differences in demographic characteristics between the two groups. However, diabetes mellitus was more common among survivors than among non-survivors. Univariate analyses showed that the amounts of daily output at 48, and 72 h, and 3-day cumulative input and output, were significantly associated with 7-day mortality risk regardless of the cumulative fluid balance (HR: 0.28, 95% CI: 0.12–0.70, *p* = 0.01 for daily output at 48 h; HR: 0.34, 95% CI: 0.13–0.85, *p* = 0.02 for daily output at 72 h.; HR: 0.72, 95% CI: 0.61–0.86, *p* = 0.01 for 3-day cumulative input; HR: 0.65, 95% CI: 0.41–0.90, *p* = 0.01 for 3-day cumulative output). Adjusted multivariate analyses showed that the lower 3-day cumulative output is an independent risk factor for 7-day and 14-day mortality.

**Conclusions:**

In our study, increased cumulative output were significantly associated with reduced short-term mortality risk in chronic hemodialysis patients undergoing CRRT regardless of cumulative fluid balance. Further prospective studies to investigate the association between fluid balance and mortality in ESRD patients requiring CRRT are warranted.

**Supplementary Information:**

The online version contains supplementary material available at 10.1186/s12882-022-02725-7.

## Background

Continuous renal replacement therapy (CRRT) is a principal intervention in critically ill patients with acute kidney injury (AKI), following admission to intensive care unit (ICU). Approximately 4% of critically ill patients with AKI require renal replacement therapy [1] and up to 80% of AKI patients undergo CRRT as the treatment modality [2]. Because critical illness at admission to ICU and a great burden of comorbidities contribute to being unable to tolerate the hemodynamic shifts of conventional hemodialysis, CRRT is frequently used also in end-stage kidney disease (ESKD) patients admitted to ICU [2].

Fluid overload is associated with worsening renal dysfunction, an increased length of ICU stay, and elevated mortality risk [3–5]. Several studies have demonstrated that an increased cumulative fluid balance is significantly associated with greater mortality risk in critically ill patients with AKI [3, 4, 6–8]. Therefore, negative fluid balance has been considered a basic approach to improve survival rates in critically ill patients with AKI.

Patients with preexisting ESKD are vulnerable to fluid overload and common causes of death are cardiovascular disease and sepsis [9, 10], which would require a large volume of intravenous fluid for initial resuscitation management. Consequently, ESKD patients with critical illness would become volume-overloaded, contradicting to current knowledge regarding fluid management in patients with AKI. Therefore, we investigated the association between fluid balance and mortality in patients with ESKD undergoing chronic hemodialysis and requiring CRRT.

## Methods

### Study design and participant

**Fig. 1:**
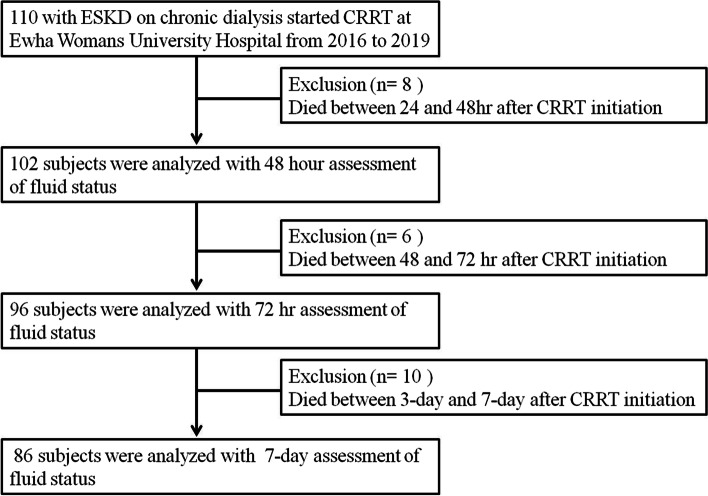
Flow diagram for patient enrollment

We retrospectively analyzed the medical records of ESKD patients treated with CRRT following initiation of chronic hemodialysis at Ewha Womans University Mokdong Hospital from January 1, 2016 to March 31, 2019. Exclusion criteria were as follows: age < 18 years, first management with renal replacement therapy, death within 24 h following CRRT initiation, and/or absence of data regarding fluid status. In patients with > 1 admission to the ICU during hospitalization, only data from the first ICU admission were analyzed. The flow diagram of patients enrolled in the study is shown in Fig. 1. In total, 110 patients were included during the study period. Of these, 8 and 6 patients died at 24–48 h and at 48–72 h after CRRT initiation, respectively. Therefore, 102 patients with 48 h assessment of fluid status, 96 patients with 72 h assessment of fluid status, and 86 patients with 7-day assessment of fluid status were analyzed. During the study period, total 46 patients died and the number of deaths were accounted for 24/46 (52%) and 34/46 (74%) within 7 days and 14 days, respectively; after 14 days from CRRT initiation, the number of deaths was sharply reduced to 39/46 (84%) within 28 days. Therefore, we arbitrarily defined 7-day and 14-day mortalities as early and late mortalities, respectively.

The study protocol was approved by the Institutional Review Board of Ewha Womans University Mokdong Hospital (IRB no. EUMC 2020–12–042). Informed consent was waived because of the retrospective nature of the study, and because the analyses used anonymous clinical data. All clinical investigations were conducted in accordance with the 2013 Declaration of Helsinki guidelines.

### Demographic and clinical measurements

All data including demographics, laboratory findings, fluid status, CRRT data, and clinical information were recorded at the time of CRRT initiation. Baseline characteristics analyzed in this study were age, sex, body mass index, comorbidities, cause of hospitalization, Charlson Comorbidity Index, Sequential Organ Failure Assessment score, Acute Physiology and Chronic Health Evaluation (APACHE) II score, Glasgow Coma scale (GCS) score, systolic blood pressure, diastolic blood pressure, and mean arterial pressure. In addition, the requirements for mechanical ventilation and vasopressor use were investigated. The prescriptions for CRRT including blood flow rate, fluid removal rate, dialysate flow rate, replacement flow rate, and use of anticoagulants were evaluated. Laboratory data were collected at CRRT initiation, while the estimated glomerular filtration rate (eGFR) was calculated using the Modification of Diet in Renal Disease equation. Sepsis was defined based on the Sepsis-II definition [11].

### CRRT prescription

The decisions to initiate and discontinue CRRT were made by nephrologists to ensure that a unified indication for CRRT could be implemented. The primary indication was hemodynamically unstable status, while secondary indications were expert opinions and clinician requests. Continuous venovenous hemodiafiltration was the standard mode of CRRT for all enrolled patients. At Mokdong Hospital, the following settings are considered standard for CRRT initiation: targeted goal effluent flow rate, 35 mL/kg/h; the pre-dilution method; blood flow rate of 150 ml/min at initiation; 1:1 ratio of replacement and dialysate flow rate. Heparin and nafamostat mesylate were used as regional anticoagulants. After CRRT initiation, clinicians monitored the actual delivered CRRT dose and continuously modified the CRRT prescription according to each patient’s condition. All patients were treated with CRRT through a newly cannulated temporary venous dialysis catheter. Common sites of insertion were femoral, internal jugular, and subclavian veins.

### Fluid assessment

Total fluid intake and output were recorded from initiation to discontinuation of CRRT. The amounts of individual and cumulative fluid input and output were assessed for 24, 48, and 72 h, as well as 3 and 7 days from CRRT initiation. The cumulative fluid balance was also calculated by subtracting cumulative output from cumulative input.

### Statistical analysis

The patients’ baseline characteristics were compared using the independent t-test for continuous variables and the chi-square test for categorical variables. Continuous variables are presented as the mean ± standard deviation, while categorical variables are presented as numbers and percentages. The study endpoint was all-cause mortality at 14 days after CRRT initiation, and patients were classified into two groups: 14-day survivors vs. non-survivors. Cox proportional hazard analyses were performed to predict 14-day mortality on the basis of the input, output, and cumulative fluid balance amounts at each endpoint. Significant covariates were identified by univariate analyses, and the clinically relevant variables were selected for multivariate analyses.

Before all investigations, standard analyses of multicollinearity and fit were applied to the regression model. The calculated variance inflation factors between cumulative input and output and cumulative fluid balance at 3 and 7 days were < 10, which is generally an acceptable range for predictors without multicollinearity. No correction for multiplicity of comparisons was performed due to the exploratory nature of the study. The results were considered statistically significant when the P value was < 0.05. Corresponding effect sizes were reported as hazard ratios with 95% confidence intervals (95% CIs). Statistical analyses were performed using the Statistical Package for the Social Sciences version 18.0 (SPSS Inc., Chicago, IL, USA).

## Results

### Baseline characteristics and clinical parameters at CRRT initiation

The baseline characteristics and clinical and laboratory parameters at CRRT initiation are shown in Tables 1 and 2. The mean age of study participants at baseline was 67.9 ± 11.4 years and 71 patients (64.5%) were male. All 110 patients were in the ICU at the time of CRRT initiation, and most (83/110, 75.5%) had sepsis. In total, 35 patients (31.8%) required mechanical ventilation at the time of CRRT initiation and more than half of the patients (52.7%) were prescribed vasopressor treatment due to hemodynamic instability. The overall rate of in-hospital mortality was 41.8% (46/110). Moreover, 69.1% of patients (76/110) survived longer than 14 days; the overall median survival time from CRRT initiation was 7.0 days. Cumulative 7-, 14-, and 28-day mortalities were 21.8%, 30.9%, and 35.5%, respectively.

**Table 1:** Baseline Characteristics

	**Overall (*n* = 110)**	**14-day survival**		***p*-value**
		Survivor, *n* = 76	Non-survivor, *n* = 34	
Demographics				
Age	67.9 ± 11.4	67.8 ± 11.1	68.2 ± 12.1	0.86
Male, *n* (%)	71 (64.5)	50 (65.8)	21 (61.8)	0.68
Body weight at admission (Kg)	60.9 ± 12.5	61.3 ± 11.8	59.8 ± 14.0	0.56
BMI at admission (kg/m^2^)	60.9 ± 12.5	23.4 ± 4.2	22.5 ± 4.0	0.31
Comorbidities, *n* (%)				
Hypertension	79 (71.8)	58 (76.3)	21(61.8)	0.12
Diabetes	61 (55.5)	47 (61.8)	14(41.2)	0.04
CVDs	46 (41.8)	34 (44.7)	12(35.3)	0.35
Malignancy	19 (17.3)	13 (17.1)	6(17.6)	0.94
Cause of hospitalization, *n* (%)				
Sepsis	83 (75.5)	55 (72.4)	28 (82.4)	0.26
non-sepsis	27 (24.5)	21 (27.6)	6 (17.6)	
CCI	6.7 ± 2.2	6.9 ± 2.3	6.3 ± 1.9	0.20
SOFA	11.3 ± 3.4	11.6 ± 3.6	10.6 ± 2.9	0.20
GCS	8.0 ± 4.0	8.6 ± 3.8	6.4 ± 4.0	0.01
APACHE II	22.4 ± 6.8	21.3 ± 6.6	24.7 ± 6.8	0.01
Mechanical Ventilation, *n* (%)	35 (31.8)	24 (31.6)	**11 (32.4)**	0.94

Data were presented as mean ± standard deviation or number (%). Abbreviation: *BMI* Body mass index, *CVD* Cardiovascular disease, *CCI* Charlson Comorbidity Index, *SOFA*, Sequential Organ Failure Assessment, *GCS* Glasgow Coma Scale, *APCHE I*I Acute Physiology and Chronic Health Evaluation II

**Table 2:** Clinical and laboratory parameters at CRRT initiation

	Overall (*n* = 110)	**14-day survival**		***p*-value**
		Survivor, *n* = 76	Non-survivor, *n* = 34	
**SBP, mmHg**	115.5 ± 24.7	118.0 ± 25.9	110.0 ± 21.1	**0.12**
**DBP, mmHg**	67.6 ± 14.5	68.5 ± 15.4	65.6 ± 12.1	**0.33**
**MAP, mmHg**	83.6 ± 14.9	85.0 ± 15.2	80.4 ± 14.0	**0.13**
**pulse rate, /min**	97.0 ± 23.4	94.5 ± 24.4	102.5 ± 20.2	**0.10**
**Vasopressor use, n (%)**	58.0 (52.7)	34 (44.7)	24 (70.6)	**0.01**
**Prescription of CRRT**				
**Duration of CRRT, day**	6.1 ± 6.7	7.0 ± 7.5	4.1 ± 3.8	**0.01**
**Fluid removal (ml/hr)**	31.5 ± 47.6	37.2 ± 49.9	18.5 ± 39.8	**0.04**
**Dialysate flow rate, mL/h**	1020.0 ± 206.8	1036.2 ± 171.8	983.8 ± 268.8	**0.22**
**Replacement flow rate, mL/h**	1170.9 ± 894.8	1225.7 ± 1058.7	1048.5 ± 273.7	**0.34**
**Blood flow rate, mL/min**	119.5 ± 30.5	120.7 ± 31.6	116.8 ± 28.1	**0.54**
**Anticoagulation use,** *n* (%)	60.0 (54.5)	48 (64)	12 (35.3)	**0.01**
**Laboratory findings**				
**Creatinine, mg/dL**	5.2 ± 2.2	5.22 ± 2.17	5.18 ± 2.27	**0.94**
**eGFR, mL/min/1.73m**^**2**^	13.3 ± 10.0	13.28 ± 10.78	13.19 ± 7.95	**0.96**
**White blood cells, n/μL**	11.7 ± 7.5	11.31 ± 6.52	12.59 ± 9.51	**0.41**
**Hemoglobin, g/dL**	9.2 ± 1.7	9.23 ± 1.59	9.03 ± 1.9	**0.57**
**Platelets, × 103/μL**	143.8 ± 84.9	142.8 ± 84.16	145.91 ± 87.63	**0.86**
**PT-INR**	2.2 ± 5.6	2.03 ± 5.76	2.61 ± 5.33	**0.64**
**Total bilirubin, mg/dL**	1.0 ± 1.1	0.91 ± 1.03	1.13 ± 1.11	**0.36**
**Aspartate aminotransferase, IU/L**	183.5 ± 918.1	168.33 ± 1024.15	217.56 ± 631.24	**0.80**
**Alanine aminotransferase, IU/L**	99.1 ± 471.5	95.54 ± 545.4	106.79 ± 250.34	**0.91**
**Lactic acid, mg/dL**	32.2 ± 39.5	26.1 ± 29.85	44.62 ± 52.88	**0.13**
**Blood gas analysis**				
**pH**	7.4 ± 0.1	7.37 ± 0.09	7.38 ± 0.11	**0.50**
**base excess**	-2.8 ± 6.3	-2.74 ± 6.26	-2.84 ± 6.54	**0.94**
**PaO2, mmHg**	104.4 ± 57.9	105.21 ± 53.39	102.48 ± 68.16	**0.82**
**PaCO2, mmHg**	42.0 ± 27.7	43.93 ± 32.49	37.4 ± 8.77	**0.27**

Data were presented as mean ± standard deviation or number (%). Abbreviation: *CRRT*, Continuous renal replacement therapy, *SBP* Systolic blood pressure, *DBP* Diastolic blood pressure, *MAP* Mean arterial pressure, *eGFR* Estimated glomerular filtration rate, *PT-INR* Prothrombin time-international normalized ratio

Patients were stratified into two groups: 14-day survivors (*n *= 76) and non-survivors (*n *= 34). There were no significant differences in demographic characteristics (e.g., age, sex, body weight, and body mass index at admission). However, diabetes mellitus was more common among survivors than among non-survivors. Sepsis was a predominant cause for hospitalization in both groups, and mechanical ventilation was performed in similar proportions of patients. There were no significant differences in the Sequential Organ Failure Assessment and Charlson Comorbidity Index scores, but higher APACHE II and lower GCS scores were identified in non-survivors. There were no significant differences in reported blood pressure at CRRT initiation, although more non-survivors had received vasopressor treatment to maintain hemodynamic stability (70.6% vs. 44.7% in non-survivors and survivors, respectively). Regarding the CRRT parameters, a significantly longer duration of CRRT and larger amount of fluid removal were prescribed among survivors, compared to non-survivors. Anticoagulants were more often prescribed among survivors. Other clinical and laboratory parameters at CRRT initiation did not significantly differ between the two groups (Table 2).

### The Comparisons of fluid balance between survivors and non-survivors

We assessed the cumulative input/output at 24, 48, and 72 h from CRRT initiation, as well as the cumulative fluid balance at 3 and 7 days after CRRT initiation (Table 3). There were no significant differences in daily inputs at 24, 48, and 72 h between survivor and non-survivor groups. Daily outputs at 48 and 72 h were significantly higher among survivors than among non-survivors, whereas there was no significant difference in output at 24 h. Compared to non-survivors, survivors had larger amounts of cumulative inputs and outputs at 3 and 7 days after CRRT initiation. There were no significant differences in cumulative fluid balance at 3 and 7 days.

**Table 3:** Comparisons of fluid balance between survivors and non-survivors

	14-day survival			
	Overall	Survivor, *n* = 76	Non-survivor, *n* = 34	*p*-value
24-h input, L	3.0 ± 2.3	3.2 ± 2.6	2.7 ± 1.2	0.31
24-h output, L	1.6 ± 2.1	1.9 ± 2.4	1.0 ± 1.1	0.05
48-h input, L	2.7 ± 1.4	2.8 ± 1.4	2.5 ± 1.1	0.25
48- hr output, L	1.8 ± 1.3	2.1 ± 1.3	1.0 ± 1.0	< 0.001
72-h input, L	2.6 ± 1.0	2.7 ± 1.1	2.3 ± 0.8	0.16
72-h output, L	2.2 ± 1.5	2.5 ± 1.5	1.2 ± 0.9	< 0.001
Cumulative input on 3 day, L	7.1 ± 4.7	7.8 ± 5.1	5.6 ± 2.9	0.02
Cumulative output on 3 day, L	4.7 ± 4.3	5.7 ± 4.5	2.3 ± 2.5	< 0.001
CFB on 3 day, L	2.5 ± 4.1	2.1 ± 4.4	3.3 ± 3.3	0.17
Cumulative input on 7 day, L*	11.1 ± 8.7	12.2 ± 9.2	8.3 ± 6.6	0.04
Cumulative output on 7 day, L*	7.9 ± 7.4	9.3 ± 7.6	4.4 ± 5.3	< 0.001
CFB on 7 day, L*	3.1 ± 5.8	2.8 ± 5.6	4.7 ± 6.3	0.21

**Data were presented as mean ± standard deviation**

^*^ Subcohort who survived 7 days or more (n = 86)

**Abbreviation: *CFB* Cumulative fluid balance**

### Effects of fluid balance on 7-day and 14-day mortalities

More than half of the deaths were within 7 days after CRRT initiation (52% of all deaths), and the mortality rate was reduced at 14 days after CRRT initiation (78% of all deaths at 14 days; 84% of all deaths at 28 days). Thus, we compared the effects of fluid balance on 7-day and 14-day mortality risks. Univariate Cox proportional hazards analyses were conducted to evaluate the effects of input/output at 24, 48, and 72 h on mortality risk. The effects of cumulative input/cumulative output and cumulative balance fluid at 3 and 7 days on mortality risk were also evaluated. Tables 4 and 5 show the crude hazard ratios (HRs) for 7-day and 14-day mortalities associated with various characteristics and amounts of input/output.

**Table 4:** The associations between clinical parameters and 7-, 14-day mortalities

	7-day mortality			14-day mortality		
Variable	HR	95% CI	*p*-value	HR	95% CI	*p*-value
Age (per 1 year)	1.00	0.97—1.04	0.93	1.00	0.97—1.03	0.82
sex (male)	0.73	0.32—1.64	0.45	0.84	0.42 -1.68	0.61
Comorbidities						
Diabetes mellitus	0.45	0.20—1.02	0.05	0.50	0.25—0.98	0.04
Hypertension	0.43	0.19—0.97	0.04	0.57	0.28—1.14	0.11
Cardiovascular disease	0.55	0.23—1.32	0.18	0.70	0.35—1.42	0.33
Malignancy	0.73	0.27—1.96	0.55	0.91	0.37—2.19	0.82
Sepsis	1.69	0.58—4.94	0.31	1.63	0.67—3.93	0.28
Vasopressor use	1.96	0.84—4.58	0.12	2.45	1.17—5.12	0.02
GCS	0.82	0.73—0.93	< 0.001	0.87	0.79—0.96	< 0.001
APACHE II	1.08	1.02—1.15	0.01	1.07	1.02—1.12	0.01
Prescription of CRRT						
Duration of CRRT	0.53	0.38—0.74	< 0.001	0.90	0.82—0.99	0.04
Fluid removal rate (mL/hr)	0.99	0.98—1.00	0.06	0.99	0.98—1.00	0.06
Use of anticoagulation	1.81	0.80—4.07	0.15	0.40	0.20—0.81	0.01

Abbreviation: *HR* Hazard ratio, *CI* Confidence interval, *CFB* Cumulative fluid balance, *APCHE II* Acute Physiology and Chronic Health Evaluation II, *GCS* Glasgow Coma Scale

**Table 5:** The associations between fluid balance and 7-,14-day mortalities

	7-day mortality			14-day mortality		
Variables*	HR	95% CI	*p*-value	HR	95% CI	*p*-value
24 h input	0.67	0.32—1.40	0.29	0.94	0.67—1.32	0.72
24 h output	0.61	0.31- 1.19	0.15	0.73	0.40—1.33	0.31
48 h input	1.06	0.70—1.60	0.79	0.83	0.45—1.55	0.56
48 h output	0.28	0.12—0.70	0.01	0.67	0.38—1.19	0.17
72 h input	0.26	0.08—0.88	0.03	0.88	0.44—1.75	0.71
72 h output	0.34	0.13—0.85	0.02	0.44	0.23—0.86	0.02
Cumulative input on 3 day	0.72	0.61—0.86	0.01	1.01	0.89—1.14	0.92
Cumulative output on 3 day	0.65	0.41—0.90	0.01	0.77	0.64—0.93	0.01
CFB on 3 day	1.08	0.96—1.21	0.21	1.07	0.96—1.20	0.24
Cumulative input on 7 day†				1.04	0.99—1.09	0.15
Cumulative output on 7 day†				1.01	0.94—1.09	0.79
CFB on 7 day†				1.08	0.98—1.18	0.11

^*^Per 1L increase

^†^ Subcohort who survived 7 days or more (*n* = 86)

Abbreviation: *HR*, Hazard ratio, *CI* Confidence interval, *CFB* Cumulative fluid balance

Preexisting diabetes was significantly associated with reduced 14-day mortality risk (HR: 0.50, 95% CI: 0.25–0.98, *p* = 0.04). A similar tendency was observed regarding 7-day mortality, although it was not statistically significant. The presence of sepsis did not influence 7-day or 14-day mortality risks. Vasopressor use was a predictor for 14-day mortality (HR: 2.45, 95% CI: 1.17–5.12, *p* = 0.02). Increased GCS and decreased APACHE II scores were associated with favorable (HR: 0.82, 95% CI: 0.73–0.93, *p* < 0.001 for GCS in 7-day mortality; HR: 1.08, 95% CI: 1.02–1.15, *p* = 0.01 for APACHE II in 7-day mortality; HR: 0.87, 95% CI: 0.79–0.96, *p* < 0.001 for GCS in 14-day mortality; HR: 1.07, 95% CI: 1.02—1.12, *p* = 0.01 for APACHE II in 14-day mortality). Regarding CRRT parameters, longer duration of CRRT and use of anticoagulants were associated with better 14-day survival outcomes. However, the fluid removal rate was not significantly predictive of 7-day or 14-day mortality risks.

Increased daily output at 48 and 72 h significantly lowered both 7-day mortality risks. Cumulative input/output at 3 day were associated with 7-day mortality, such that more fluid input led to better survival outcomes (Table 5). Daily input/output and cumulative input at 3 day, and cumulative input/output at 7 day did not affect 14-day mortality, whereas output at 72 h and cumulative output at 3 day were significantly associated with decreased 14-day morality risk (HR: 0.44, 95% CI: 0.23–0.86, *p* = 0.02 for 72 h output; HR: 0.77, 95% CI: 0.64–0.93, *p* = 0.01 for cumulative output at 3 day). However, there were no significant associations between cumulative fluid balance at 3 and 7 days and mortality risk.

Subsequently, multivariate Cox regression analyses were conducted. The hazard ratios of fluid balance were calculated after adjustment for age, sex, diabetes mellitus, vasopressor use, CRRT duration, anticoagulant use, and GCS and APACHE II scores, all of which were significant confounders in the crude model. After stepwise adjustment (model 1, adjusted for age, sex, history of diabetes, and hypertension; model 2, additional adjustment for vasopressor use, GCS and APACHE II scores, anticoagulant use, and CRRT duration; Table 6), increased cumulative output at 3 day significantly reduced the 7- and 14-day mortality risks, but the corresponding cumulative inputs were not. In the multivariate analysis of 14-day mortality, cumulative input/output at 7 day were not associated with mortality risk.

**Table 6:** The adjusted hazard ratio of cumulative input and output for 7-, 14-day mortalities

	7-day mortality				14-day mortality			
	Model 1		Model 2		Model 1		Model 2	
	HR (95% CI)	*p*-value	HR (95% CI)	*p*-value	HR (95% CI)	*p*-value	HR (95% CI)	*p*-value
Cumulative input on 3 day	0.96 (0.79—1.17)	0.71	0.85 (0.68—1.06)	0.53	1.01 (0.90—1.13)	0.92	0.98 (0.75—1.03)	0.78
Cumulative output on 3 day	0.67 (0.48—0.93)	0.01	0.66 (0.47—0.93)	0.04	0.78 (0.64—0.94)	0.01	0.79 (0.65—0.98)	0.03
					Model 1		Model 2	
					HR (95% CI)	*p*-value	HR (95% CI)	*p*-value
Cumulative input on 7 day†					1.05 (0.97—1.14)	0.20	1.03 (0.94—1.13)	0.50
Cumulative output on 7 day†					0.84 (0.79—1.02)	0.09	0.91 (0.80—1.03)	0.14

^*^per 1.0 L increase

^†^ Subcohort who survived 7 days or more (*n* = 86)

Model 1: Adjusted for age, sex, history of diabetes and hypertension

Model 2: Adjusted for age, sex, history of diabetes and hypertension, use of vasopressor, Glasgow Coma Scale, Acute Physiology and Chronic Health Evaluation II scale, and duration of CRRT

Abbreviation: *HR* Hazard ratio, *CI* Confidence interval

### Effects of fluid balance on 7-day and 14-day mortalities, following input and output stratification

Patients were divided into low and high cumulative input/output groups by median split to evaluate the effects of input and output on mortality (Table 7). High levels of cumulative input and output at 3 days were associated with reduced 7-day mortality risk, whereas 14-day mortality risk was related to the cumulative output at 3-days alone. We examined whether input and output had a combined effect on mortality risk. The patients were stratified into four groups according to the amounts of cumulative input and output at 3 and 7 days, and the group with combined low input and low output was used as the reference (Table 8). The group with high input and high output had reduced hazard ratios compared to the high input/low output and low input/high output groups, although the differences were not statistically significant.

**Table 7:** The comparisons of 7-, 14-day mortalities associated with cumulative input/output by median split

	7-day mortality		14-day mortality	
	HR (95% CI)	*p*-value	HR (95% CI)	*p*-value
High cumulative input on 3 day (vs. low cumulative input)	0.81 (0.24—0.97)	0.04	1.25 (0.55—3.05)	0.67
High cumulative output on 3 day (vs. low cumulative output)	0.18 (0.04—0.85)	0.03	0.35 (0.14—0.89)	0.02
High cumulative input on 7 day (vs. low cumulative input)			1.30 (0.48—3.53)	0.60
High cumulative output on 7 day (vs. low cumulative output)			0.75 (0.29—1.94)	0.55

Abbreviation: *HR* Hazard ratio, *CI*, Confidence interval

**Table 8:** The association between low cumulative input/output and high input/output for 7-,14-day mortality risks

	7- day mortality				14-day mortality			
	HR (95% CI)	*p*-value	HR (95% CI)	*p*-value	HR (95% CI)	*p*-value	HR (95% CI)	*p*-value
Cumulative fluid on 3 day	Low output		High output		Low output		High output	
Low input	Reference		0.31 (0.35—2.74)	0.30	Reference		0.72 (0.17—3.00)	0.64
High input	1.49 (0.37—5.68)	0.56	0.16 (0.02—1.45)	0.10	2.49 (0.81—7.63)	0.11	0.50 (0.13—1.85)	0.30

Abbreviation: *HR* Hazard ratio, *CI* Confidence interval

## Discussion

ESKD patients admitted to the ICU may have low effective arterial blood volume and hemodynamic instability due to various combinations of sepsis, cardiac dysfunction, and other underlying diseases. Restoration of intravascular volume and circulation may require the administration of large volumes of intravenous fluids. Moreover, fluid overload (e.g., pulmonary edema, respiration failure, and/or third-spacing) may be present on ICU admission in ESKD patients on dialysis who are vulnerable to poor fluid balance. Thus, clinicians have chosen CRRT as a dialysis option to reduce fluid overload and secure hemodynamic stability. However, it remains unclear how to manage volume status by controlling CRRT input and output in these patients. Although a number of literatures have been dealing with this issue, most studies have focused on AKI patients. To our knowledge, the present study is the first attempt to investigate fluid balance in ESKD patients undergoing chronic hemodialysis and requiring CRRT.

The main findings of our study were as follows. First, early mortality (i.e., 7-day mortality) was associated with early fluid management, such that 3-day cumulative output produced better survival outcomes in ESKD patients undergoing CRRT. Following adjustment for confounders, the relationship between early mortality and early cumulative output remained statistically significant. Second, early fluid management reduced late mortality risk (i.e., 14-day mortality) with a significant effect of 3-day cumulative output.

Our results were consistent with some, but not all, previous reports. In the PICARD study, among critically ill patients with AKI, survivors who required renal replacement therapy had significantly lower levels of fluid accumulation at initiation and cessation of dialysis than non-survivors, despite adjustments for dialysis modality and severity score [4]. Subgroup analyses of the RENAL study concluded that a negative fluid balance during CRRT was associated with an improved survival outcome in critically ill patients with AKI [12]. Specifically, a number of studies have directly examined the association between cumulative fluid balance and mortality among AKI patients receiving CRRT. Ostermann et al. reported that a decline of cumulative fluid balance was associated with both ICU mortality and hospital mortality, whereas cumulative fluid balance at CRRT initiation was not independently associated with mortality [13]. Ryu et al. postulated that early cumulative fluid balance was significantly associated with increased early (7-day) and late (28-day) mortality risks. Moreover, increases in 24- and 72-h cumulative fluid balance were significantly associated with increases in 7-day and 28-day mortality regardless of cumulative input. The authors of that study suggested that the impact of cumulative fluid balance on mortality may be more dependent on cumulative output [14]. Although their study population consisted of patients with sepsis requiring CRRT, Uusalo et al. showed that higher fluid balance during first 72 h of CRRT was independently associated with hospital mortality in patients with AKI, even after adjustment for repeated measures of disease severity over the period when CRRT was required [15]. Among the patients with AKI, hospital survivors had a significantly lower cumulative fluid balance at CRRT initiation compared to non-survivors. The repeated time-dependent net fluid balance during the first 72 h after CRRT initiation was associated with mortality in the univariate counting process model. Even after careful adjustment for repeated measures of disease severity, the association between net fluid balance during the first 72 h of CRRT and hospital mortality remained significant in critically ill patients with AKI. In our study, greater cumulative input and output volumes at 3 days rather than cumulative fluid balance were significantly associated with better 7-day mortality outcomes. In the comparisons of mortality risk, in which patients were stratified into low and high cumulative input/output groups on day 3, the group with high input and low output tended to have higher 7- and 14-day mortality risks compared to the reference group (i.e., low input/output) (Table 8). These observations suggested that fluid balance of cumulative high input and cumulative low output, referred to as positive fluid balance, was related to the increased mortality risk seen in ESKD patients undergoing CRRT, similar to previous studies [13–15]. However, our study lacked information on volume status at CRRT initiation in ESKD patients and the actual amount of fluid overload over the period when CRRT was required, which limited our ability to compare of mortality risk with previous studies.

Notably, cumulative input was suggested to yield survival benefits in our study. In univariate analysis, increases in both cumulative input and output at 3 days were significantly associated with reduced hazard ratios for 7-day mortality (Table 5). In addition, in comparisons of mortality risk, the group with high input and high output tended to have lower 7- and 14-day mortality risks compared to the reference group (i.e., low input/output) (Table 8). These results should be interpreted cautiously given the observational design of our study. As a large amount of fluid was administered for initial resuscitation, it may have seemed as though there was an increase in cumulative input at 3 days, even though the amount of fluid administered was reduced when hemodynamic stability was achieved. Moreover, the surviving patients who tolerated the initial round of resuscitation recovered and ultimately exhibited successful cumulative input/output in the later period. Naturally increased cumulative input may have led to a survival benefit in the analysis. Therefore, rather than unconditionally increasing the input and output, it is necessary to titrate the input according to the patient’s needs and ability to tolerate fluid removal, while simultaneously considering the corresponding increase in output.

Our results conflict with the findings of some previous studies. Notably, there were no differences in survival outcome according to fluid management between sepsis and non-sepsis patients. Dialysis patients are particularly susceptible to infections due to uremia-related immune deficiency, defective phagocytic function, old age, comorbidities (e.g., diabetes mellitus), and anatomical abnormalities (e.g., polycystic kidney disease), as well as repetitive exposures to nosocomial microorganisms during the normal course of dialysis therapy [16–18]. Vascular access for hemodialysis including the dialysis catheter, arteriovenous fistula, and graft increases the risk for infectious complications [19]. ESKD patients undergoing hemodialysis reportedly have higher sepsis-related mortality rates, compared to the general population, despite adjustments for age, ethnicity, and diabetes status [20]. In the present study, however, sepsis was not a predictor for short-term mortality in ESKD patients requiring CRRT. Patients who died within 24 h immediately after CRRT initiation were excluded from the study; thus, the mortality rate from sepsis might have been underestimated. Moreover, ESKD itself is an independent predictor for mortality in patients with sepsis [21], and therefore the impact of sepsis on mortality might have been masked by the likelihood that patients have concomitant life-threatening conditions (e.g., hyperkalemia and metabolic acidosis).

In our study, non-survivors had significantly higher APACHE II scores, compared to survivors; the difference in APACHE II scores between sepsis and non-sepsis patients was not statistically significant (Supplementary Table 1). Moreover, the survival outcomes were unaffected by age and sex, whereas previous studies have shown associations of older age and male sex with adverse outcomes. Notably, the presence of uncomplicated acute respiratory failure requiring mechanical ventilation was not related to mortality risk in this study. The integrated effect of clinical profile at the time of CRRT initiation was presumably more important for predicting mortality, rather than a single evaluation parameter.

There were several limitations to this study. First, it was retrospective and conducted at a single center with a relatively small sample size. Thus, selection bias was not completely excluded, and the results may not be generalizable to patients of other ethnicities. Regardless, we minimized bias throughout the study by our statistical analysis approach. Second, the baseline fluid balance prior to commencement of CRRT was not available for the analysis. Initial volume status on admission to ICU or CRRT initiation would be important factors related CRRT outcomes [22], but limited data has impeded the precise understanding of the relationship between fluid balance and mortality in ESKD patients requiring CRRT. Third, we investigated the relationship between fluid balance and short-term mortality but not other outcomes (e.g., hospital mortality or 1-year mortality). Accordingly, a long-term follow-up study is needed to clarify the effects of fluid management. Finally, the study design was non-interventional, and the observed association between fluid balance and outcome does not constitute a causal relationship.

## Conclusion

Our study highlighted the importance of early fluid management during CRRT in chronic hemodialysis patients undergoing CRRT. Increased cumulative output was significantly associated with reduced early mortality risk in chronic hemodialysis patients undergoing CRRT regardless of cumulative fluid balance. Further studies are necessary to confirm the associations between fluid balance and mortality in chronic dialysis patients requiring CRRT.

## Supplementary Information

**Additional file 1. **

## Data Availability

The data that support the findings of this study are available from the corresponding author upon reasonable request.

## Abbreviations

CRRT: Continuous renal replacement therapy
ESKD: End-stage kidney disease
CVD: Cardiovascular disease
BMI: Body mass index
CCI: Charlson Comorbidity Index
SOFA: Sequential Organ Failure Assessment
GCS: Glasgow Coma Scale
APCHE II: Acute Physiology and Chronic Health Evaluation II
SBP: Systolic blood pressure
DBP: Diastolic blood pressure
MAP: Mean arterial pressure
eGFR: Estimated glomerular filtration rate
PT-INR: Prothrombin time-international normalized ratio
CFB: Cumulative fluid balance
HR: Hazard ratios
CI: Confidence intervals
