# Supplementary material for: Early fluid management affects short-term mortality in patients with end-stage kidney disease undergoing chronic hemodialysis and requiring continuous renal replacement therapy
Source: BMC Nephrol. 2022 Mar 14;23:102. doi: 10.1186/s12882-022-02725-7 (PMC8919557; doi:10.1186/s12882-022-02725-7)
Supplement: Supplementary file 1 — Additional file 1. [file 12882_2022_2725_MOESM1_ESM.docx]

Supplementary Table 1. Comparisons of clinical scale based on presence of sepsis

|  | Non-sepsis | Sepsis | *p*-value |
| --- | --- | --- | --- |
| CCI | 5.26 ± 2.19 | 7.20 ± 1.97 | 0.85 |
| SOFA | 11.38 ± 3.45 | 11.23 ± 3.39 | 0.86 |
| GCS | 8.00 ± 3.98 | 7.94 ± 4.02 | 0.95 |
| APACHE II | 22.70 ± 7.09 | 22.27 ± 6.78 | 0.77 |

Data were presented as mean ± standard deviation.

Abbreviation: CCI, Charlson Comorbidity Index; SOFA, Sequential Organ Failure Assessment; APCHE II, Acute Physiology and Chronic Health Evaluation II; GCS, Glasgow Coma Scale
